# Biorelevant *In Vitro* Skin Permeation Testing and *In Vivo* Pharmacokinetic Characterization of Lidocaine from a Nonaqueous Drug-in-Matrix Topical System

**DOI:** 10.1208/s12249-021-02101-y

**Published:** 2021-08-12

**Authors:** Emileigh Greuber, Kip Vought, Kalpana Patel, Hiroaki Suzuki, Kazuhiro Usuda, Akira Shiramizu, Luana Pesco Koplowitz, Barry Koplowitz, Howard I. Maibach, Dmitri Lissin

**Affiliations:** 1 Scilex Pharmaceuticals Inc., 960 San Antonio Road, Suite 100, Palo Alto, California 94303 USA; 2 Oishi Koseido Co., Ltd., Pharmaceutical Development Center, Tosu, Japan; 3 Duck Flats Pharma, Flemington, New Jersey USA; 4 Dermatology Department, University of California San Francisco, San Francisco, California USA

**Keywords:** lidocaine, *in vitro* permeation test (IVPT), pharmacokinetics, TS (topical system)

## Abstract

Recently, lidocaine topical systems utilizing nonaqueous matrices have been developed and provide efficient lidocaine delivery through the skin, such that lower concentrations of drug provide equivalent or greater drug delivery than drug-in-matrix hydrogel lidocaine patches. This study characterizes drug delivery from a nonaqueous lidocaine topical system with increasing drug load both *in vitro* and *in vivo*. Topical systems formulated with either 1.8% or 5.4% lidocaine were applied to healthy volunteers’ backs (*n* = 15) for 12 h in a single-center, open-label, four-treatment, four-period crossover pharmacokinetic study. Subjects were dosed with either three 1.8% systems or one, two, or three 5.4% systems in each period. Blood was collected for up to 48 h, and plasma lidocaine levels were measured with a validated HPLC method. In parallel, human and mouse skin models characterized the *in vitro* skin permeation profile. The pharmacokinetic profile was linear between one, two, and three lidocaine 5.4% applications. Application of three lidocaine 1.8% systems (108 mg lidocaine) was bioequivalent to one lidocaine 5.4% system (108 mg lidocaine). Both topical systems remained well adhered to the skin and irritation was mild. The 5.4% system had approximately threefold higher skin permeability than the 1.8% system in the mouse and human skin models. The results indicate increasing the drug load by three times results in triple the drug delivery both *in vivo* and *in vitro*. The relationship between the *in vitro* permeation and *in vivo* absorption correlates and is nonlinear.

## INTRODUCTION

Analgesic topical systems, commonly referred to as patches, are increasingly used for the treatment of localized pain (1). In contrast to other routes of administration, such as oral, topical drug delivery via an adhesive patch can provide drug directly to the targeted tissue for a sustained period of time, avoid gastrointestinal and hepatic first-pass metabolism, and reduce side effects associated with high systemic exposures (2). Skin functions as a formidable barrier to the outside environment by protecting from physical insults, chemicals, microbes, and allergens. The stratum corneum, the outermost layer of skin, is composed of a 10–15-μm-thick matrix of dehydrated and dead keratinocytes, embedded in lipid layers, and is critical in skin barrier formation (3, 4). Topical analgesics must overcome this barrier for drug penetration to occur to reach the site of action at the nerves in the dermis. Ideal characteristics of topical/transdermal drugs include low molecular weight (<500 Da), sufficient aqueous and lipid solubility (logP between 1 and 3), and sufficiently low melting point (<200°C) (5–7).

The amide anesthetic agent lidocaine is a suitable candidate for topical administration for pain treatment (8–10). Lidocaine is a weak base (pKa 8.01) with a molecular weight of 234 Da and is relatively lipophilic (logP of 2.44) (11). When applied to the skin, it can diffuse through the stratum corneum to damaged nerves, where it blocks voltage-gated sodium channels expressed on Aδ and C fibers (12).

In 1999, the US Food and Drug Administration (FDA) approved Lidoderm® (Endo Pharmaceuticals, Malvern, PA), a 5% prescription lidocaine patch, for the treatment of pain associated with post-herpetic neuralgia (PHN) (13). In 2018, FDA approved ZTlido® (Scilex Pharmaceuticals, Palo Alto, CA), a 1.8% prescription topical system, also for the treatment of PHN pain (14). While both products are passive patch diffusion systems, Lidoderm® only delivers ~3% of its drug load through the skin in contrast to ZTlido®, which delivers ~50% of its drug load. ZTlido® is formulated with less drug than Lidoderm® (36 mg/140 cm^2^ system *vs*. 700 mg/140 cm^2^ patch), and the products provide bioequivalent lidocaine exposure (15). The improved bioavailability of ZTlido® relative to Lidoderm® is due to formulation differences. ZTlido® utilizes a nonaqueous formulation, where lidocaine is dissolved in organic acid and polyalcohol, which keeps lidocaine soluble and available for percutaneous penetration (16), whereas Lidoderm® is a hydrogel formulation where lidocaine is dissolved in a water-soluble polymer (17). While the 1.8% concentration system provides equivalent exposure to Lidoderm® for the treatment of PHN, this nonaqueous adhesive formulation is adaptable to increasing drug load and may be able to provide more lidocaine through the skin, which potentially could be useful in the treatment of painful neuropathic or musculoskeletal conditions.

To characterize the effect of increasing drug load on both skin permeability and systemic bioavailability, a triple strength (5.4%) version of ZTlido® was developed and compared to the original 1.8% strength using *in vitro* skin permeation test (IVPT) studies with hairless mouse skin and human abdominal skin, and an *in vivo* pharmacokinetic study was conducted in healthy human volunteers.

## MATERIALS AND METHODS

### Materials

Lidocaine topical system 5.4% (Lot 30901A) and ZTlido® (lidocaine topical system 1.8%, Lot 190142 and 17810A) from Scilex Pharmaceuticals Inc. (Palo Alto, CA) were used as test and reference products. Lidocaine USP reference standard was from Moehs Catalana SRL. All reagents used for the preparation of receptor medium and for high-performance liquid chromatography (HPLC) were of analytical grade.

### *In Vitro* Skin Permeation Studies

#### *In Vitro* Permeability Studies Across Hairless Mouse Skin

Frozen hairless mouse skin from three 7-week-old female Hos:HR-1 mice was obtained from Hoshino Laboratory Animals, Inc. (Japan) and kept frozen until use. Lidocaine systems were cut into 1.5 cm × 1.5 cm squares and applied to the stratum corneum side of thawed hairless mouse skin, which was mounted on a side-by-side diffusion type cell (diffusion area: 0.6 cm^2^) maintained at 32.0 ± 0.5°C, with the skin placed between the donor and receptor cells. Isotonic Sörensen buffer (5 mL; 67 mM sodium phosphate at pH 7.4, 75 mM sodium chloride) was added to the receptor cell. Receptor cell fluid samples (100 μL) were collected at 2, 4, 8, and 12 h. The same volume of fresh receptor fluid was reinjected into the chamber after each sampling. All receptor solution samples were analyzed by HPLC.

#### *In Vitro* Permeability Studies Across Human Post-Surgical Skin

Human skin from three individuals (donors) was collected during abdominal surgical procedures and stored frozen (−20°C) until use. Thawed skin was separated from any remaining hypodermis and cut to fit onto a 2-cm^2^ Franz-type glass diffusion cell. The skin was mounted onto the diffusion cell with a 3-mL receptor compartment with the epidermis facing the donor compartment. The receptor compartment contained Sörensen buffer (pH 7.4) supplemented with 50 μg/mL gentamycin sulfate. Skin surface temperature was maintained at a target of 32°C to model normally *in vivo* skin surface temperature using a temperature-regulated water jacket around the receptor compartment heated to 37°C. The barrier integrity of each skin piece was tested by measuring transepidermal water loss using Tewameter TM300 (Courage & Khawazaka Electronic GmbH, Germany) at times 0, 12, and 24 h after topical system application. Any skin piece with a transepidermal water loss measurement greater than 9 g/m^2^/h was excluded.

The topical systems were applied to the top of the skin mounted on a diffusion cell for 12 h, after which time they were removed, and the skin remained mounted onto the diffusion cell for another 12 h. In addition to the topical systems, lidocaine in DMSO solution (154 μg/mL) was also tested as a positive control. Lidocaine in DMSO solution was not removed or wiped off after 12 h. Receptor fluid samples (1 mL) were collected every 60 min for the first 8 h and then at 12 h and 24 h. The same volume of fresh receptor fluid was reinjected into the chamber after each sampling. All receptor solution samples were analyzed by HPLC.

#### Determination of Lidocaine in Receptor Fluid

In the mouse study, lidocaine determination in receptor fluid was performed by a validated reverse-phase HPLC with UV detection method at 230 nm using Xterra® RP15 5μm, 15 × 3.9 mm chromatographic column and KH_2_PO_4_ buffer/acetonitrile (3:2 v/v) as mobile phase with a 0.9 mL/min flow rate and injection volume of 20 μL. In the human study, lidocaine determination was performed by a reverse-phase UHPLC method at 230 nm using Acquity BEH C18 1.7 μm, 50 × 2.1 mm chromatographic column using the same mobile phase as the mouse method with a 0.7 mL/min flow rate and injection volume of 4 μL.

#### Data Analyses

For the mouse and human studies, the mean and standard deviation of cumulative lidocaine permeation were calculated. Maximum flux values at steady state (J_max_, expressed in μg/cm^2^/h) were calculated by determining the slope of the linear portion of each graph:

$$ {\mathrm{J}}_{\mathrm{max}}=\frac{Qcum(t2)- Qcum(t1)}{t2-t1} $$

where *Qcum* (*t*1) is the cumulative permeated lidocaine at time point *t*_1_ (μg/cm^2^) and *Qcum* (*t*2) is the cumulative permeated at time point *t*_2_ (μg/cm^2^), and *t*1 and *t*2 are the time points (h).

In the human study, statistical results were analyzed with GraphPad Prism (GraphPad Software, Inc., La Jolla, CA, USA). Variability within and between donors was evaluated for cumulative lidocaine permeation amounts in receptor fluid at 24 h, using a two-way ANOVA test on ln-transformed data to verify that replicates could be treated as independent values (no donor effect). Statistical analysis was performed using a one-way ANOVA (*n* = 12 individual values per formulation) on ln-transformed data followed by a Tukey’s multiple comparisons test.

### *In Vivo* Study

#### Subjects

Fifteen healthy, nonsmoking, adult male and female volunteers were enrolled. The study protocol was approved by IntegReview Institutional Review Board (Austin, TX, USA) and conducted in accordance with *Good Clinical Practice* and the Declaration of Helsinki. Eligible subjects were men and women 18–60 years of age with BMI between 18 and 32.5 kg/m^2^, nonsmokers, and generally healthy as documented by 12-lead electrocardiogram and clinical laboratory assessments. Female subjects could not be pregnant or lactating, and those of childbearing potential were instructed to practice medically acceptable contraception throughout the study. Exclusion criteria included evidence of allergy or known hypersensitivity to lidocaine, local anesthetics of the amide type, or any of the components of the lidocaine topical system formulation. Subjects were excluded if they had any major illness in the last 3 months or any significant chronic medical illness, history of addiction, and abuse or misuse of any drug or had any skin condition that may affect the application of the study product. The study was conducted at Axis Clinicals (Dilworth, MN, USA) and is registered with ClinicalTrials.gov, NCT04819581.

#### Study Design

This was a single-center, open-label, randomized, four-treatment, four-sequence, four-period, single-dose crossover pharmacokinetic (PK) study to compare the single-dose PK of lidocaine 1.8% *vs*. either 1, 2, or 3 applications of lidocaine 5.4%.

**Fig. 1:**
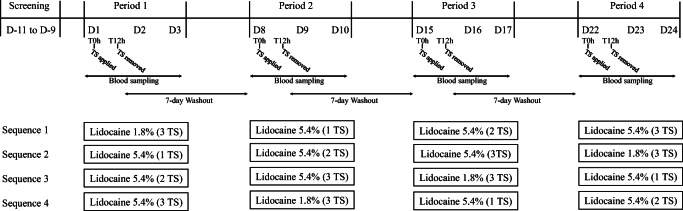
Schematic of pharmacokinetic study design. Randomized, four-treatment, four-sequence, four-period healthy volunteer study to compare the single-dose PK of lidocaine 1.8% *vs*. either 1, 2, or 3 applications of lidocaine 5.4% (TS: topical system)

Subjects remained in the clinical facility overnight before administration of the topical systems and were discharged 24 h post-dose. As per the randomization schedule, either 1, 2, or 3 of lidocaine 5.4% topical systems and 3 lidocaine 1.8% topical systems were applied to the subjects’ back. The washout time between periods was 7 days. All subjects received snacks and meals at appropriate times as not to interfere with the product application or other study activities. A schematic of the study design is in Fig. 1.

#### Blood Sampling

Blood samples (6 mL aliquots) were collected by venipuncture in vacutainer tubes containing K_2_EDTA at time 0 (before topical system application) and at 2.0, 4.0, 6.0, 9.0, 10.0, 11.0, 12.0, 13.0, 14.0, 16.0, 18.0, 20.0, 22.0, 24.0, and 48.0 h in each study period. Centrifugation separated plasma and plasma samples were frozen until analysis.

#### Determination of Lidocaine in Plasma Samples

Lidocaine determination in receptor fluid was performed by a validated LC-MS/MS method using Thermo® Aquasil C18, 100 × 2.1 mm chromatographic column, and Sciex API 4500 mass spectrometer. The method’s lower limit of quantitation was 0.2000 ng/mL.

### Pharmacokinetic Analysis

Lidocaine pharmacokinetic parameters were calculated using drug concentration-time data by noncompartmental method with Phoenix WinNonlin® Software, Version 8.0 (Certara USA, Inc.). Descriptive statistics were computed and reported for primary (C_max,_ AUC_0-t_, AUC_0-∞_) and secondary parameters (t_max_, K_el_, t_1/2_, apparent dose, V_d_/F, and CL/F) for each treatment for each subject in each study period. ANOVA was computed for untransformed and ln-transformed PK parameters of C_max,_ AUC_0-t_, AUC_0-∞_ for lidocaine. All statistical analyses were performed using the mixed effect ANOVA model (PROC MIXED) of SAS® Release 9.4 software (SAS Institute Inc., Cary, NC, USA).

### *In Vitro–In Vivo* Comparison

The plasma concentration data of lidocaine from all subjects and treatments were individually deconvoluted using the unit impulse response pharmacokinetic parameters as reported by Dyck *et al*. (18). The unit impulse response was a three exponential function derived from the NONMEM values for IV bolus administration, which was then extrapolated to a 1-mg IV bolus. A validated WinNonlin v8.2 software was used to perform the deconvolution. The dimensions of both product strengths (1.8% and 5.4%) were 10 × 14 cm, resulting in an application surface area of 140 cm^2^ per topical system. The amount of lidocaine per topical system was 36 mg for the 1.8% strength and 108 mg for the 5.4% strength. Results from the deconvolution analysis were calculated at the same time points used in the *in vitro* permeation experiment, which were 0, 1, 2, 3, 4, 5, 6, 7, 8, 12, and 24 h. Each individual plasma lidocaine concentration-time curve was deconvoluted after which the averages and standard deviations were calculated.

### Adhesion Analysis

Product adhesion was assessed immediately after application and at 3, 6, 9, and 12 h, just prior to product removal. Adhesion was assessed by a trained scorer using a transparent grid with evenly spaced dots. The grid was demarcated to the size of the product and was gently laid over the product on the skin and areas of adhesion outlined; dots excluded from adhering areas were counted to determine the total product adhesion as a percentage (percent adhesion).

### Irritation Analysis

Application site skin irritation was evaluated 30 min and 2 h after product removal using an 8-point dermal response scale recommended by the FDA (19), where 0 represents no evidence of irritation and 7 represents a strong reaction, extending beyond the application site, and a scale of other effects, including glazed appearance, peeling and cracking, dried or serous exudates covering at least a portion of the application site, and small petechial erosions and/or scabs.

## RESULTS

To examine the effect of drug load on the skin permeability of a nonaqueous topical system, lidocaine formulations containing either 1.8% or 5.4% were manufactured. Their characteristics are outlined in Table I. Both products were formulated with the same adhesive base with increasing lidocaine concentration using the same hot melt manufacturing process. The adhesive was thinly layered onto a nonwoven polyester backing material and covered with a polyethylene terephthalate release liner. The finished product size (10 cm × 14 cm) and adhesive mass were the same for each with minor differences in thickness.

**Table I:** Characteristics of Lidocaine 1.8% and Lidocaine 5.4% Topical Systems

Attribute	Lidocaine 1.8%	Lidocaine 5.4%
Topical system size	10 cm × 14 cm (140 cm^2^)	10 cm × 14 cm (140 cm^2^)
Topical system thickness	0.8 mm	1.0 mm
Lidocaine content	36 mg	108 mg
Adhesive mass	2 g	2 g
Adhesive type	Polyisobutylene/styrene-isoprene-styrene block copolymer	
Other inactive ingredients	Butylated hydroxytoluene, dipropylene glycol, isostearic acid, mineral oil, silicon dioxide, terpene resin, and nonwoven polyester cloth backing	

### *In vitro* Mouse Skin Permeation

**Fig. 2:**
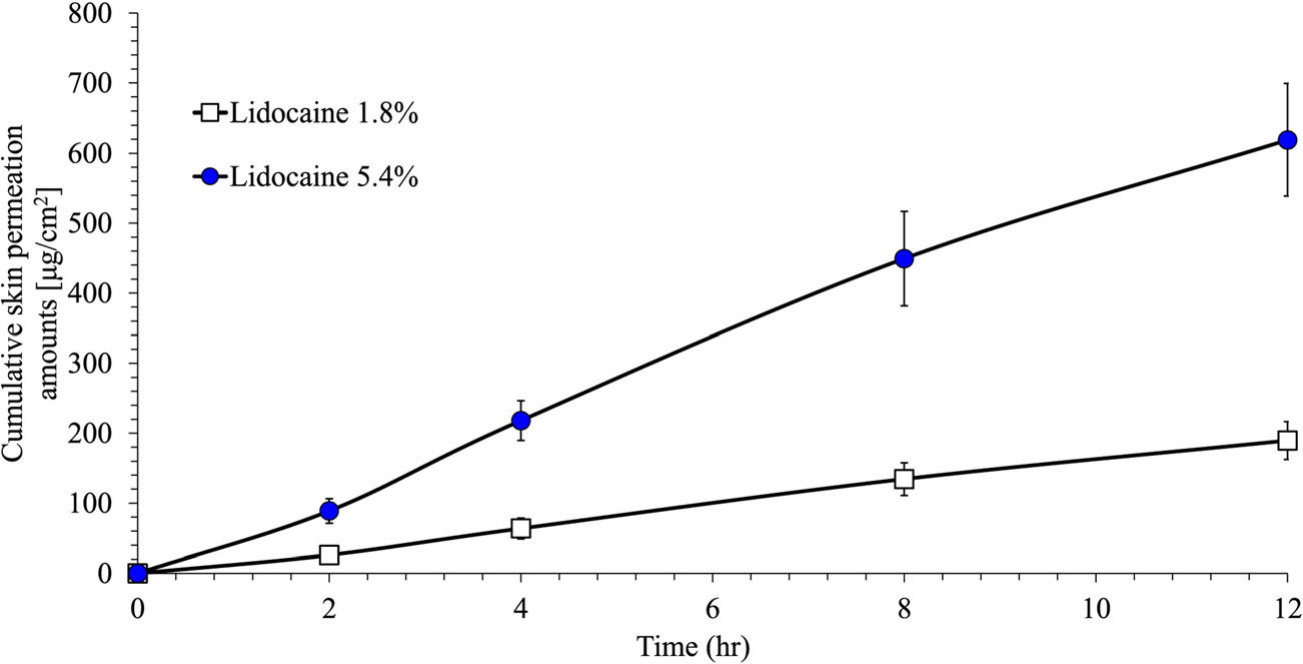
Cumulative lidocaine permeation through mouse skin (means ± SD of *n* = 3)

To assess how the change in drug load affected skin permeability, a nude mouse model was selected. Nude mouse skin is less than half as thick as human skin and is more permeable than human skin to many drugs (20) and thus was selected as a sensitive model to screen whether formulation differences affect permeability. Lidocaine 1.8% and 5.4% systems were applied to full-thickness mouse skin placed on a side-by-side glass diffusion cell for 12 h. Cumulative lidocaine permeation is presented in Fig. 2 and Table II. After 12 h, the permeation of lidocaine 5.4% was ~3-fold higher than the lidocaine 1.8% system (619.08 ± 80.28 μg/cm^2^ *vs*. 189.47 ± 27.16 μg/cm^2^). The maximum flux at steady state was also approximately 3-fold higher (53.13 μg/cm^2/^/h *vs*. 16.41 μg/cm^2/^/h).

**Table II:** Descriptive Statistics of Mean Skin Permeation Parameters in Mouse Skin

Formulation	*N*	Cumulative lidocaine (μg/cm^2^) (±SD)	J_max_ (μg/cm^2^/h) (±SD)
Lidocaine 1.8%	3	189.47 (27.16)	16.41 (1.90)
Lidocaine 5.4%	3	619.08 (80.28)	53.13 (6.68)
Ratio of lidocaine 5.4%:lidocaine 1.8%		3.27	3.24

### *In vitro* Human Skin Permeation

**Fig. 3:**
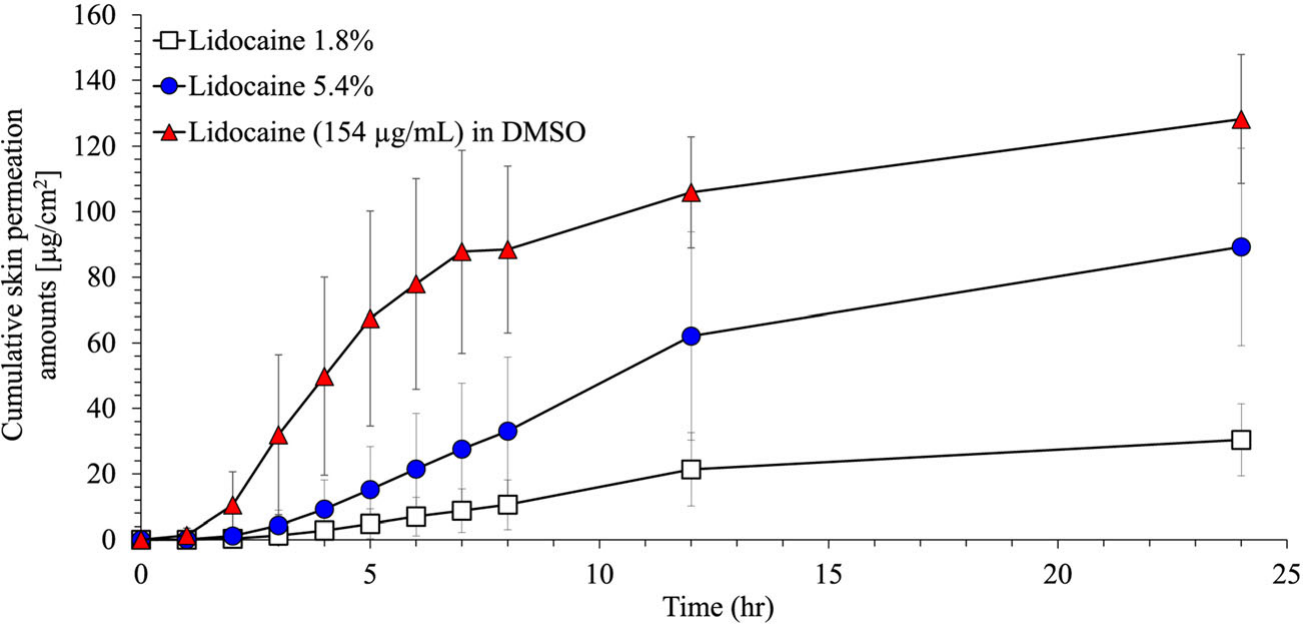
Cumulative lidocaine permeation through full-thickness human skin (means ± SD of *n* = 3)

To assess whether the higher lidocaine permeability observed in the mouse model was also observed in human skin, an IVPT study was conducted with full-thickness, post-surgical abdominal skin from three donors (Table III). Topical systems were applied to human skin and placed on a vertical static Franz cell for 12 h. After 12 h, the systems were removed, and lidocaine in the receptor fluid was monitored for an additional 12 h. In addition to the topical systems, a solution of lidocaine dissolved in dimethyl sulfoxide (DMSO) was tested as a positive control, given that DMSO is a well-established skin permeation enhancer for lidocaine (21, 22). Permeation profiles of all three formulations were significantly different from one another (Table IV and Fig. 3). The cumulative amount of permeated lidocaine at 24 h was statistically different between lidocaine at 1.8% and 5.4% (*p* < 0.0001). Lidocaine 5.4% delivered approximately 3-fold more lidocaine than lidocaine 1.8%. The DMSO solution (which contained the same amount of lidocaine as the lidocaine 5.4% topical system) delivered 1.4-fold more lidocaine after 24 h compared to the lidocaine 5.4% (*p* = 0.0117) and about 4.2-fold compared to lidocaine 1.8% (*p* < 0.0001).

**Table III:** Demographic Information for the *In Vitro* Study Skin Donors and *In Vivo* PK Study Subjects

	*In vitro* (*N* = 3)	*In vivo* (*N* = 15)
**Age in years**		
Mean (± SD) Range	45.7 25–70	42.07 ± 11.90 25–58
**Sex,** ***n*** **(%)**		
Male Female	1 (33.3%) 2 (66.6%)	10 (66.7%) 5 (33.3%)
**Ethnicity,** ***n*** **(%)**		
Black Caucasian Asian American Indian/Alaska Native	0 3 (100%) 0 0	4 (26.6%) 9 (60.0%) 1 (6.7%) 1 (6.7%)
**BMI in kg/m**^**2**^		
Mean (± SD) Range		25.97 ± 3.72 20.74–31.85
**Thickness in mm**		
Mean (± SD) Range	1.33 ± 0.29 0.90–2.03

**Table IV:** Descriptive Statistics of Mean Skin Permeation Parameters in Human Skin

Formulation	*N*	Cumulative lidocaine (ng/cm^2^) (±SD)	J_max_ (μg/cm^2^/h) (±SD)
Lidocaine 1.8%	12	30,399 (11,041)	2.1 (1.1)
Lidocaine 5.4%	12	89,247 (30,106)	6.1 (3.0)
Lidocaine (154 μg/mL) in DMSO	11	128,278 (19,623)	15.5 (4.9)
Ratio of lidocaine 5.4%:lidocaine 1.8%		2.94	2.90

### Pharmacokinetic Study of Lidocaine in Humans

Fifteen subjects were randomized, and all received treatment. Fourteen (14) subjects completed the study; one was discontinued after the first period. Overall, subjects ranged from 25 to 58 years of age, and the majority were white (60.0%) (Table III). All subjects were healthy, with vital signs in the normal range.

In this four-treatment, four-sequence, four-period, single-dose crossover PK study, either one, two, or three lidocaine 5.4% systems or three lidocaine 1.8% systems were applied to intact skin on the back of the volunteers for 12 h (Fig. 1). This design was chosen to assess the linearity between one, two, and three applications of the higher strength and to compare PK of the higher strength to the maximum daily dose of lidocaine 1.8% (3 topical systems) according to its FDA-approved label (14).

**Fig. 4:**
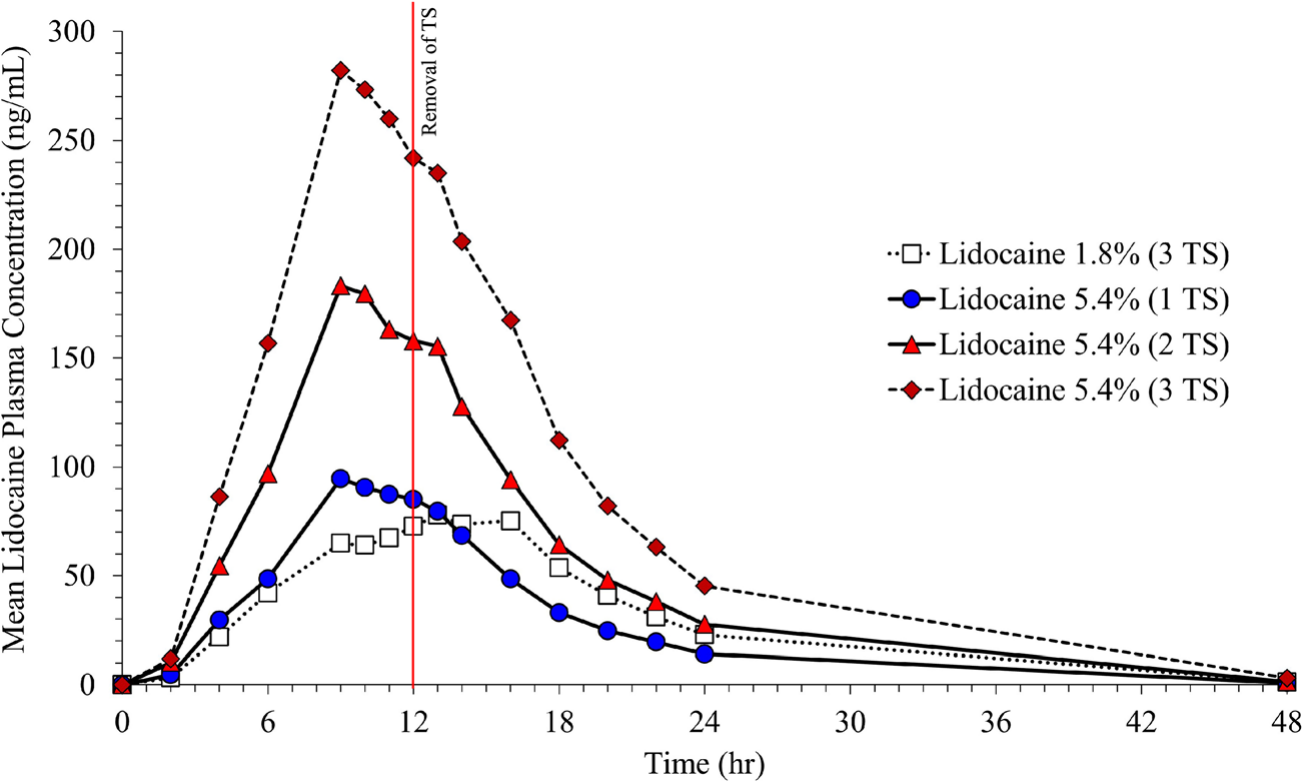
Mean lidocaine plasma concentration *vs*. time curve in healthy human volunteers

**Fig. 5:**
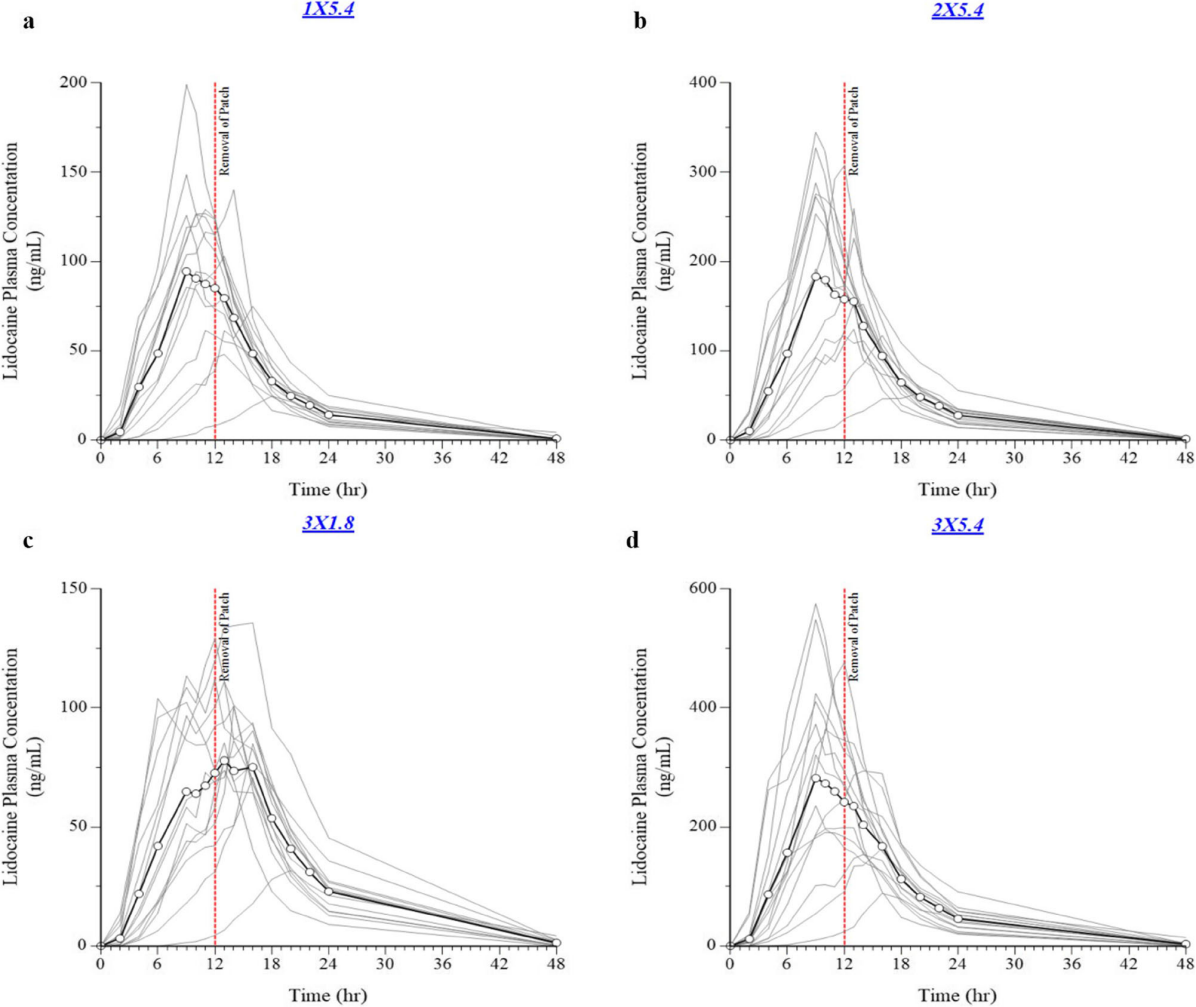
Individual lidocaine plasma concentration *vs*. time curves. Grey lines are the individual subject’s concentration time curve, and heavy black lines with circles are the mean concentration time curves for **a** lidocaine 5.4% (1 TS), **b** lidocaine 5.4% (2 TS), **c** lidocaine 1.8% (3 TS), and **d** lidocaine 5.4% (3 TS)

Application of three lidocaine 1.8% systems resulted in a C_max_ of 93.6± 26.5 ng/mL, AUC_0-∞_ of 1406.83 ± 464.94, and a median t_max_ of 13.50 h. The PK profile of one lidocaine 5.4% system was similar, with a C_max_ of 106.25 ± 45.5 ng/mL, AUC_0-∞_ of 1260.60 ± 398.56, and slightly shorter median t_max_ of 11.0 h (Table V, Fig. 4). Application of one, two, or three 5.4% systems was linear with respect to C_max_, AUC_0-∞_, and AUC_0-t_ (Table V, Fig. 4). The t_max_ was similar for all 5.4% systems, with the median between 10 and 12 h, and was slightly shorter than that observed for the 1.8% system (13.50 h), although there was considerable variability between individuals. Spaghetti plots of each individual’s PK profile by treatment are in Fig. 5. As expected, no differences were observed in lidocaine half-life, elimination constant, and apparent volume of distribution or apparent clearance between treatments (Table V).

**Table V:** Descriptive Statistics of Pharmacokinetic Parameters

Paramter (unit)	Lidocaine 1.8% (3 TS) *N* = 14	Lidocaine 5.4% (1 TS) *N* = 14	Lidocaine 5.4% (2 TS) *N* = 14	Lidocaine 5.4% (3 TS) *N* = 15
C_max_ (ng/mL)	93.65 ± 26.46	106.25 ± 45.5	222.86 ± 92.17	320.93 ± 149.06
AUC_0-t_ (ng·h/mL)	1388.67 ± 471.33	1242.68 ± 408.92	2447.25 ± 803.18	3939.13 ± 1469.21
AUC_0-∞_ (ng·h/mL)	1406.83 ± 464.94	1260.60 ± 398.56	2459.40 ± 796.55	3971.40 ± 1479.33
t_max_ (h)*	13.50 [6.00, 20.00]	11.00 [9.00, 18.00]	12.00 [9.00, 20.00]	10.00 [9.00, 16.00]
K_el_ (h^−1^)	0.13 ± 0.03	0.14 ± 0.04	0.14 ± 0.02	0.13 ± 0.03
t_1/2_ (h)	5.57 ± 1.56	5.65 ± 2.62	5.19 ± 1.04	5.60 ± 1.61
Apparent dose (mg)	46.93 ± 9.87	43.84 ± 12.73	85.79 ± 28.42	130.20 ± 41.50
V_D_/F (L)	286.69 ± 132.29	293.69 ± 157.89	265.99 ± 78.65	271.26 ± 74.43
CL/F (L/h)	35.33 ± 8.59	36.27 ± 9.43	35.84 ± 8.61	34.43 ± 8.73

*Median (range)

Systemic lidocaine exposure was also estimated by measuring the amount of residual lidocaine in used topical systems, liners, envelopes, and surface of the skin and subtracting the total residual amount from the amount of lidocaine in an unused topical system. The total mean residual amount of lidocaine recovered was 19 mg/topical system (of the possible 36 mg) for the 1.8% strength and 64 mg for the 5.4% strength (of the possible 108 mg). As shown in Table V, 46.93 ± 9.87 mg of lidocaine was absorbed from three lidocaine topical system 1.8%, for an estimated bioavailability of 43 ± 9%, compared to 43.84 ± 12.73 mg of lidocaine from one lidocaine topical system 5.4%, for an estimated bioavailability of 41 ± 12%.

As the total drug load was the same for three 1.8% systems as one 5.4% system (108 mg lidocaine), bioequivalence was assessed between these treatments per FDA guidance (23). The geometric means of the systemic rate of exposure (AUC_0-t_, AUC_0-inf_) and the extent of absorption (C_max_) were similar, and their 90% CIs were within the predefined bioequivalence range of 80% to 125% (Table VI).

**Table VI:** Analysis of Bioequivalence Between 3× Lidocaine 1.8% (108 mg) and 1× Lidocaine 5.4% (108 mg)

Parameter	*N*	Ratio % (3× lidocaine 1.8% *vs*. 1× lidocaine 5.4%) (90% confidence interval)
C_max_	14	108.38 (97.80–120.11)
AUC_0-t_	14	90.28 (83.98–97.06)
AUC_0-∞_	14	90.61 (83.89–97.85)

In addition to characterizing the PK profile, this study also assessed product adhesion and irritation. Both products remained >98% adhered throughout the study (Table VII). After removal, irritation was assessed as mild, with no irritation scores greater than 2 (Table VII). No serious adverse events, premature withdrawals due to safety, or replacements were observed. All lab results, vital signs, and post-study examination were in a normal range and did not indicate any clinical abnormality.

**Table VII:** Adhesion Performance and Skin Irritation in the Pharmacokinetic Study

Treatment	Adhesion			Irritation			
	*N* (no. of TS)	Mean % adhesion (SD)	Range mean % adhesion (min, max)	30 min after TS removal		2 h after TS removal	
				Mean irritation (SD)	Range irritation score (min, max)	Mean irritation (SD)	Range irritation score (min, max)
Lidocaine 1.8% (3 TS)	42	98.56 (2.02)	89.36, 100.00	0.6 (0.70)	0, 2	0.4 (0.54)	0, 2
Lidocaine 5.4% (1 TS)	14	99.20 (0.69)	98.02, 100.00	1.0 (0.78)	0, 2	0.7 (0.73)	0, 2
Lidocaine 5.4% (2 TS)	28	99.30 (0.78)	99.59, 100.00	1.0 (0.88)	0, 2	0.9 (0.83)	0, 2
Lidocaine 5.4% (3 TS)	45	98.56 (2.33)	88.69, 100.00	1.0 (0.80)	0, 2	0.7 (0.67)	0, 2

### *In Vitro* and *In Vivo* Relationship

**Fig. 6:**
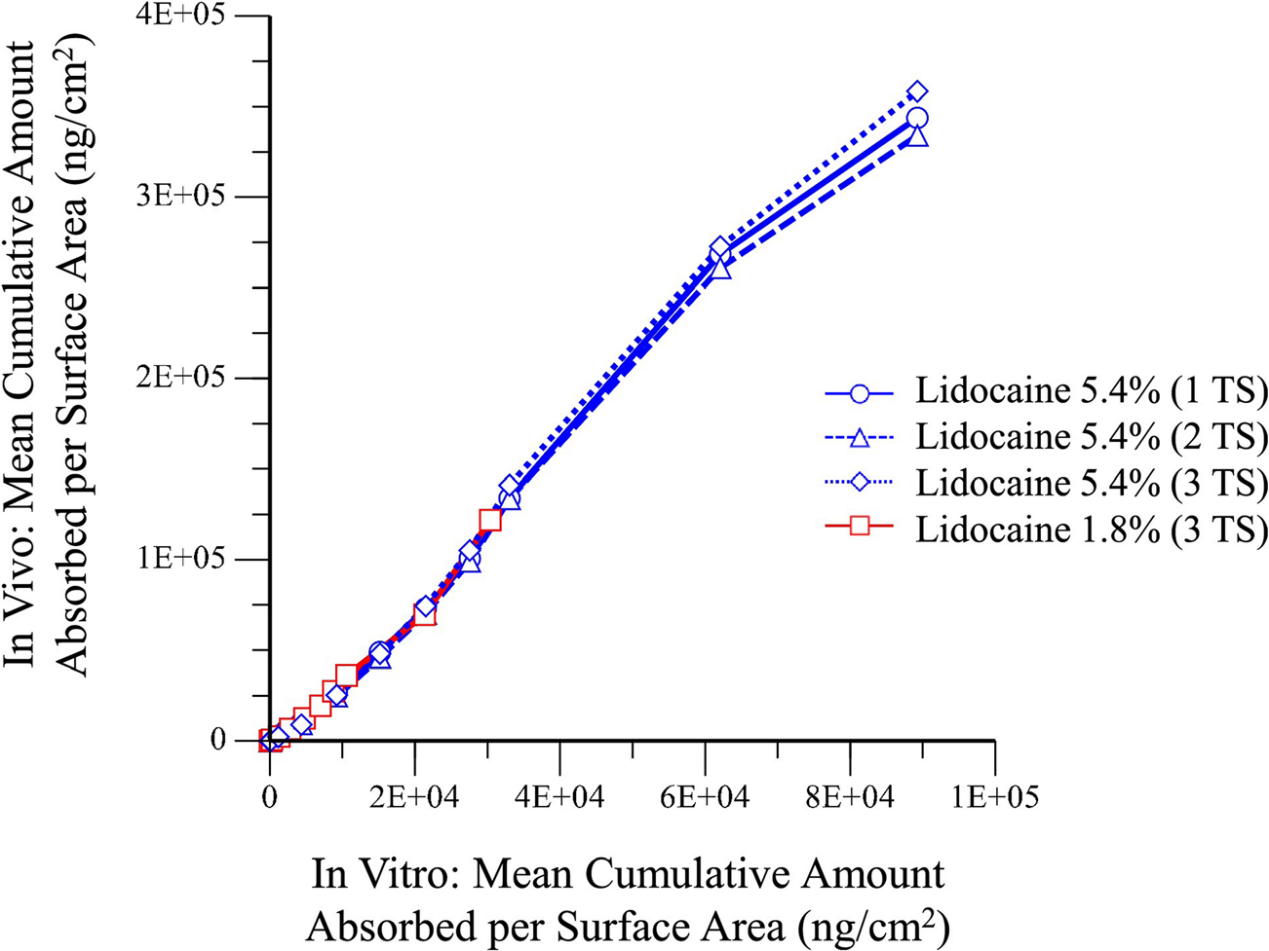
*In vivo vs*. *in vitro*: *in vivo* cumulative mean amount of lidocaine absorbed per applied surface area (ng/cm^2^) *vs*. *in vitro* cumulative mean skin permeation results

To determine if the lidocaine *in vitro* permeation findings were correlated to the *in vivo* systemic absorption, plots of the *in vivo* findings *vs*. the *in vitro* findings were generated using the same unit of measurement (ng/cm^2^). The resulting plot (Fig. 6) showed a high degree of overlap between all treatments. The relationship between *in vivo* and *in vitro* appears to be nonlinear. The data were fitted to a cubic equation:

*In vivo* cumulative amount = −5.52E-10x^3^ + 6.60E-05x^2^ + 2.38x − 531.

## DISCUSSION

Since the first transdermal scopolamine patch was approved by FDA in the 1970s, innovation in transdermal and topical system formulation development has led to better skin permeation and drug delivery (24). Here, we show permeation of lidocaine through the skin can be increased using a nonaqueous delivery system above the levels previously approved by FDA for the prescription lidocaine patch used to treat pain associated with PHN. While an attractive feature of topical delivery systems is the limited systemic exposure, this higher strength system still results in relatively low systemic exposure; an intravenous bolus of lidocaine followed by continuous infusion for the treatment of cardiac arrhythmia typically yields therapeutic plasma levels in the range of 1500–5000 ng/mL (25). The exposure presented by this higher 5.4% strength system (~106 ng/mL for one system or ~321 ng/mL for three systems) is well below these levels.

Our work also shows that *in vitro* skin permeation models can be predictive of pharmacokinetics in humans. The mouse IVPT study showed the higher strength system had approximately 3-fold higher permeation. Similarly, the human IVPT study showed a 2.9-fold increase in lidocaine permeation with the higher strength system. The *in vivo* PK study showed that applying three 1.8% systems was bioequivalent to one 5.4% system in terms of C_max_, AUC_0-t_, and AUC_0-∞_. The relationship between the *in vitro* skin permeation and *in vivo* absorption of lidocaine after topical system application was demonstrated reproducible for the various 5.4% treatments and applicable even when including the 1.8% formulation. The relationship between the *in vitro* permeation and *in vivo* systemic absorption correlates and is nonlinear in nature.

This study also shows that both products were well tolerated, with no serious adverse events or discontinuations related to adverse events. Mild skin irritation was observed upon product removal, but it resolved and was not considered significant. Both products demonstrated good adhesion to the skin, consistent with previously published reports on the 1.8% strength (15, 26, 27).

There were limitations to our study. The *in vitro* skin permeation studies had a small number of donors, and the skin all came from the same anatomical site. It is not known how anatomical site differences may affect the skin permeation of these products. The human PK study also involved a relatively small number of participants (*n* = 15), and the products were only applied to the skin on the back. There were no elderly subjects in the PK study; the oldest subject was 58 years old. Older patients may have distinct skin properties (texture, thickness, moisture) that could affect lidocaine absorption.

Lidocaine is a potent sodium channel blocker that has a well-documented effect on pain (10). Topical administration offers a convenient and noninvasive drug delivery option with limited systemic exposure and associated side effects. This study shows that it is possible to manufacture a product that can deliver more lidocaine through the skin than other prescription lidocaine topical systems on the market. The higher penetration observed both *in vivo* and *in vitro* suggests that this advanced formulation of lidocaine may provide more efficient skin penetration and potentially better efficacy.

## CONCLUSION

In this comparative bioavailability study, the rate and extent of lidocaine penetration from the skin was approximately threefold higher for the 5.4% strength lidocaine topical system as compared to the 1.8% system. This higher skin permeability may translate into clinical superiority over currently marketed prescription lidocaine topical systems for the treatment of localized pain.
